# Visualization-based discovery and analysis of genomic aberrations in microarray data

**DOI:** 10.1186/1471-2105-6-146

**Published:** 2005-06-13

**Authors:** Chad L Myers, Xing Chen, Olga G Troyanskaya

**Affiliations:** 1 Lewis-Sigler Institute for Integrative Genomics, Princeton University, Carl Icahn Laboratory, Princeton, NJ 08544, USA; 2 Department of Computer Science, Princeton University, Princeton, NJ 08544, USA; 3 Department of Computer Science, Stanford University, Stanford, CA 94305, USA

## Abstract

**Background:**

Chromosomal copy number changes (aneuploidies) play a key role in cancer progression and molecular evolution. These copy number changes can be studied using microarray-based comparative genomic hybridization (array CGH) or gene expression microarrays. However, accurate identification of amplified or deleted regions requires a combination of visual and computational analysis of these microarray data.

**Results:**

We have developed ChARMView, a visualization and analysis system for guided discovery of chromosomal abnormalities from microarray data. Our system facilitates manual or automated discovery of aneuploidies through dynamic visualization and integrated statistical analysis. ChARMView can be used with array CGH and gene expression microarray data, and multiple experiments can be viewed and analyzed simultaneously.

**Conclusion:**

ChARMView is an effective and accurate visualization and analysis system for recognizing even small aneuploidies or subtle expression biases, identifying recurring aberrations in sets of experiments, and pinpointing functionally relevant copy number changes. ChARMView is freely available under the GNU GPL at .

## Background

Aneuploidies (chromosomal copy number changes) constitute a key mechanism in cancer progression [1,2] and play important evolutionary roles in speciation [3] and adaptive mutation in yeast and microbial populations [4,5]. Array-based comparative genomic hybridization (array CGH) has enabled fast genome-wide investigations of copy-number changes [6,7]. However, once microarray experiments have been performed, accurate identification of amplifications and deletions requires a combination of manual discovery through data visualization and sophisticated statistical analysis.

Computational methods can use additional data sources, such as gene expression, to facilitate the discovery and analysis of genomic aberrations. This is possible because the presence of amplifications or deletions of whole or partial chromosomes can have substantial effects on gene expression in the affected regions [8-10]. Gene expression microarray data can serve both as a second source of information for aneuploidy detection and perhaps as an indication of which genomic changes are most functionally relevant since mRNA transcript abundance more directly affects cellular phenotype than genomic DNA content. Therefore, an effective visualization and analysis system for aneuploidy detection should make use of both array CGH and gene expression data, and allow easy examination of overlaps in the corresponding data sets.

Existing visualization tools include Caryoscope [11], CGHAnalyzer [12], Java Treeview's Karyoscope [13], and SeeCGH [14]. All of these were developed specifically for the analysis of array CGH data and with the exception of CGHAnalyzer, none allow convenient visualization of multiple experiments. Additionally, while they all offer a number of useful approaches to visualization, none include automatic statistical prediction to complement manual discovery of amplifications and deletions (see Table 1 for a detailed comparison of features of our software as compared to those of existing applications). To facilitate discovery of genomic aberrations from microarray data, novel methodology is required that integrates visualization with sophisticated statistical analysis and enables visualization of multiple experiments and data types simultaneously.

**Table 1:** Comparison with existing visualization and analysis software. Comparison of ChARMView's functionality with existing visualization and analysis software including Caryoscope [11], CGHAnalyzer [12], Java Treeview's Karyoscope [13], SeeCGH [14], CGH-Explorer [24], CGH-PRO [25], and CGH-Miner [26].

**Feature**	**ChARM View**	**Caryo-scope**	**TreeView (Karyosco pe)**	**SeeGH**	**CGH Analyzer**	**CGH Explorer**	**CGH PRO**	**CGH Miner**
**Platform**	most platforms (Java-based)	most platforms (Java-based)	most platforms (Java-based)	Windows	most platforms (Java-based)	most platforms (Java-based)	Linux, Windows	Windows, Unix, Excel add-in
**Software availability**	freely downloadab le with registration	freely downloadable	freely downloadable	freely downloadab le with registration	freely downloadable with registration	freely downloadable with registration	freely downloadab le	freely downloadable with registration
**Source-code license**	GNU GPL	MIT license	GNU GPL	not available	freely downloadable	freely downloadable	GNU GPL	freely downloadable
**External software dependencies**	none	none	none	requires MySQL database	none	none	MySQL, R	R
**Automatic statistical determination of single-array aberrations**	Yes	No	No	No	No	Yes	Yes	Yes
**Statistical analysis of manually selected regions**	Yes	No	No	No	Yes	Yes	No	No
**Simultaneous display of multiple experiments**	Yes	No	No	No	Yes	Yes	Yes	Yes
**Statistical analysis of aberrations occurring in multiple experiments**	No	No	No	No	Yes	No	No	No
**Aberration breakpoints/statistics export**	Yes	No	No	No	Yes	Yes	Yes	Yes
**Image export**	Yes	Yes	Yes	Yes	Yes	Yes	Yes	Yes
**Command-line statistical analysis feature**	Yes	No	No	No	No	No	No	No
**Allows user-defined genomic feature annotation**	Yes	Yes	Yes	Yes	Yes	Yes	Yes	Yes
**Web-linked genomic feature annotation**	No	Yes	Yes	Yes	Yes	Yes	Yes	No

Here we describe ChARMView – an integrated system that combines statistical analysis with effective visualization capabilities to enable interpretation of microarray data for aneuploidy discovery. Our system facilitates both manual and automated discovery of genomic aberrations from microarray data and can display multiple experiments and data types simultaneously. ChARM-View can be used to identify amplifications and deletions from array CGH or gene expression data independently or simultaneously, making it a powerful approach for identifying real and functionally relevant chromosomal changes.

## Implementation

ChARMView was implemented in Java using Swing set components to ensure cross-platform compatibility. Many of the visualization features were developed using the Open Source 2D graphics toolkit Piccolo developed at the University of Maryland [15].

## Results and Discussion

### Methodology: statistical analysis

ChARMView computational analysis automatically detects regions of non-random spatial bias and is appropriate for any genomic data associated with chromosomal coordinates. Statistical analysis is based on our algorithm ChARM (Chromosomal Aberration Region Miner), described in detail in [16]. ChARM identifies potential breakpoints by a differential filter followed by an accurate expectation-maximization approach. The statistical significance of each identified region is evaluated with a one-sample sign test and a permutation-based mean test. By their formulation, the significance tests are valid for any size segment, but do lose power with decreasing segment size. ChARM has been evaluated on gene expression and array CGH data: it is robust and accurate for regions as small as 4–5 probes, and sensitive enough to detect aneuploidies even in mixed populations of cells [16].

**Figure 1:**
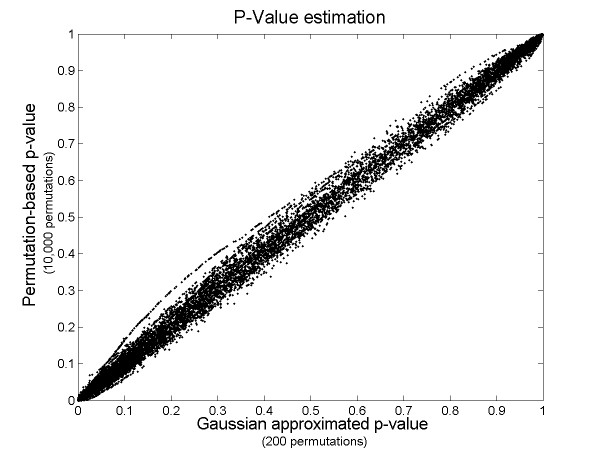
**Gaussian approximation of the permutation-based p-value. **ChARMView performs 200 permutations to assess statistical significance of predictions, then uses Gaussian approximation to estimate the p-value. The p-values based on 200 permutations and Gaussian approximation show .9991 correlation to the p-values based on 10,000 permutations as computed by ChARM.

As a system for dynamic and real-time data analysis and visualization, ChARMView requires very fast statistical algorithms. However, the permutation-based test as originally described in Myers *et al*. [16] requires non-trivial computation since it involves performing several thousand permutations of the chromosome order. To speed up the mean permutation test for the software system, we have developed an accurate approximation that requires many fewer permutations. The original version of the test requires computing the mean of the region of interest and comparing this with the means of similar-sized segments in randomly permuted data. We have verified that means of typical chromosomal segments in array CGH and gene expression data can generally be reasonably approximated with a normal distribution. This is a generally well-accepted claim even for small groups (~10) unless the underlying population is extremely non-normal, which is typically not the case for log-transformed array CGH or gene expression data. The statistical significance of predicted aneuploidy region in ChARMView is obtained by computing means of 200 permutations of chromosome ordering of the actual data, estimating the parameters, and then integrating the tail of the underlying distribution beyond the observed value. Figure 1 illustrates the correlation between p-values generated from 10,000 random permutations and p-values obtained from a normal approximation whose parameters were estimated with only 200 permutations. This approximation yields the precision of several thousand permutations based on significantly less computation. Completing a fully automated statistical analysis on a typical gene expression dataset (6000 genes over 16 chromosomes, measured in 16 experiments) requires approximately 7 seconds/experiment for a total of less than 2 minutes on a Pentium 4 3.2 GHz desktop. ChARMView also allows users to manually select regions to test for statistical significance.

### Methodology: visualization-based analysis

The most powerful aspect of ChARMView is integration of computational analysis with visualization. This combination of visualization and analysis enables users to view automated predictions of aneuploidies as well as analyze statistical significance of manually selected regions. Visualization is a critical complement to computational analysis as human perception can often identify subtle trends in the data that cannot be detected with purely computational methods. This is especially critical when comparing results of multiple experiments or experimental replicates, such as in cancer studies where researchers often search for recurring aneuploidies in a set of patients. ChARMView facilitates such discovery with visualization of multiple experimental replicates, experiments, and data types.

**Figure 2:**
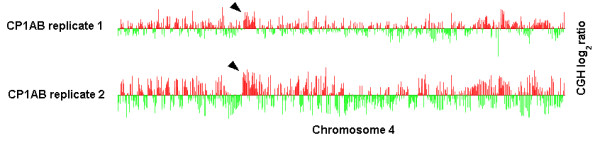
**Simultaneous visualization of replicate experiments. **A set of replicate array CGH experiments from Dunham *et al*. [5] displayed with ChARMView (chromosome 4 of CP1AB, replicates 1 and 2 shown). The region identified by the arrows is hard to distinguish from noise in either of the replicates when viewed separately, but is clearly a region of positive bias when the replicates are viewed together. This is confirmed by statistical analysis.

The most common way to increase confidence in results of an experiment is to produce replicate microarray experiments. Data from such replicate experiments is usually averaged for computational analysis. However, viewing such replicates simultaneously is an effective approach to analysis, as people are often perceptive of subtle but repeated trends that are difficult to capture with a statistical test. This visualization-based approach does not make any assumptions, such as independence assumption of the typically used Fisher meta-analysis test [17]. Thus, aligning corresponding chromosomal data from several replicates of the same experiment typically allows the user to spot trends that might otherwise go unnoticed. Figure 2 illustrates this phenomenon with two replicates of the same array CGH experiment.

**Figure 3:**
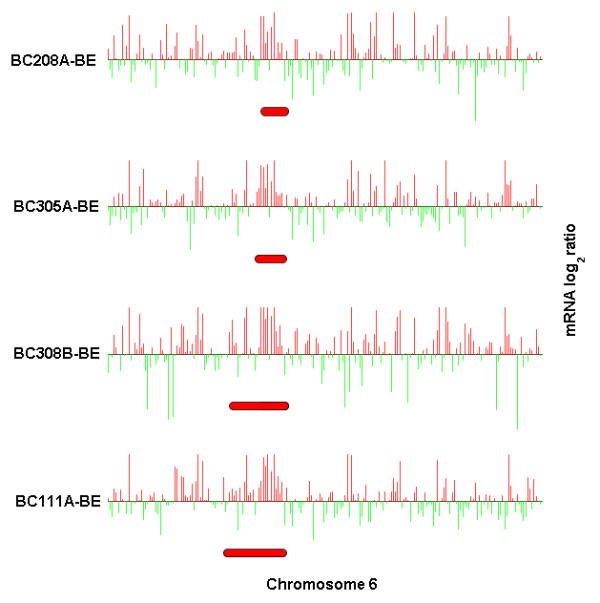
**Simultaneous visualization of multiple independent expression microarrays. **Simultaneous visualization of overlapping significant expression biases in a set of four independent breast tumor samples from Sorlie *et al*. [19] (chromosome 6 of breast tumor expression profiles BC208A-BE, BC305A-BE, BC308B-BE, and BC111A-BE shown). Each red bar below the data indicates a predicted aberration identified independently on the corresponding experiment.

The simultaneous display feature of ChARMView is also useful for visual analysis of computational prediction results for multiple experiments. This is an effective method for identifying common genomic aberrations in otherwise uncorrelated experiments or a characteristic aberration in a set of samples with a common phenotype. For example, a set of breast cancer samples [18,19] can share the same bias in gene expression that corresponds to a predicted aneuploidy or a localized expression bias, as shown in Figure 3. Overlapping predictions serve as independent confirmations that the predicted aberration is real. Furthermore, results of such analysis of multiple samples can then be used to correlate specific chromosomal aberrations with phenotypic or clinical parameters.

**Figure 4:**
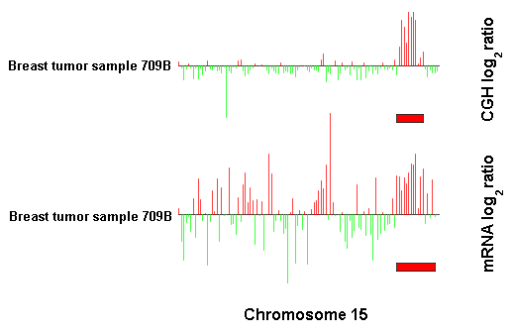
**Identifying functionally relevant genomic aberrations. **A small amplification evident in the array CGH data breast cancer data (top) [18], and its effect on mRNA expression (bottom) [19] (chromosome 15 of breast tumor sample 709B shown).

As array CGH techniques become more widely applied, the generation of copy number data is rarely the end goal of biological studies. Instead, a key challenge is deciphering which parts of a karyotypic profile are responsible for particular phenotypes. While sophisticated statistical and computational methods will certainly be required to answer these questions, the most effective approaches will also need to harness the power of human visual perception. To address this issue, ChARMView can display and analyze both array CGH and gene expression microarray data and display these diverse data and predictions for corresponding chromosomes simultaneously. Simultaneous display of array CGH and gene expression data enables researchers to observe the effect that amplification or deletion of particular sequences of genomic DNA has on the abundance of mRNA transcripts (Figure 4). We have noted a number of cases where large amplifications or deletions result in no detectable change in gene expression. These regions may be less likely to cause a particular phenotype than aneuploidies that result in drastic changes in gene expression. ChARMView facilitates convenient discovery of these changes, focusing further experimental investigation.

A final unique characteristic of ChARMView is that its visualization and statistical tools are developed for general use, independent of data type and organism. Any dataset with features that can be associated with chromosomal position can be imported and analyzed with ChARMView. For instance, the software has been particularly useful in identification of aneuploidies based on gene expression datasets although array CGH is the typical experimental approach for probing genomic amplifications or deletions. ChARMView has also been used to identify spatially-correlated biases in gene expression that are unrelated to altered chromosome structure. Generally, our tool can be used to identify any region of non-randomness with respect to position in genomic data with inherent ordering. In addition to its usefulness for a variety of data types, ChARMView can be applied to a variety of organisms. By default, the system provides chromosomal coordinates for *Saccharomyces cerevisiae *data with ORF identifiers and human data with Unigene identifiers. However, any data that can be mapped to a set of linear chromosomes can be imported and analyzed by ChARMView.

### Illustration of application

**Figure 5:**
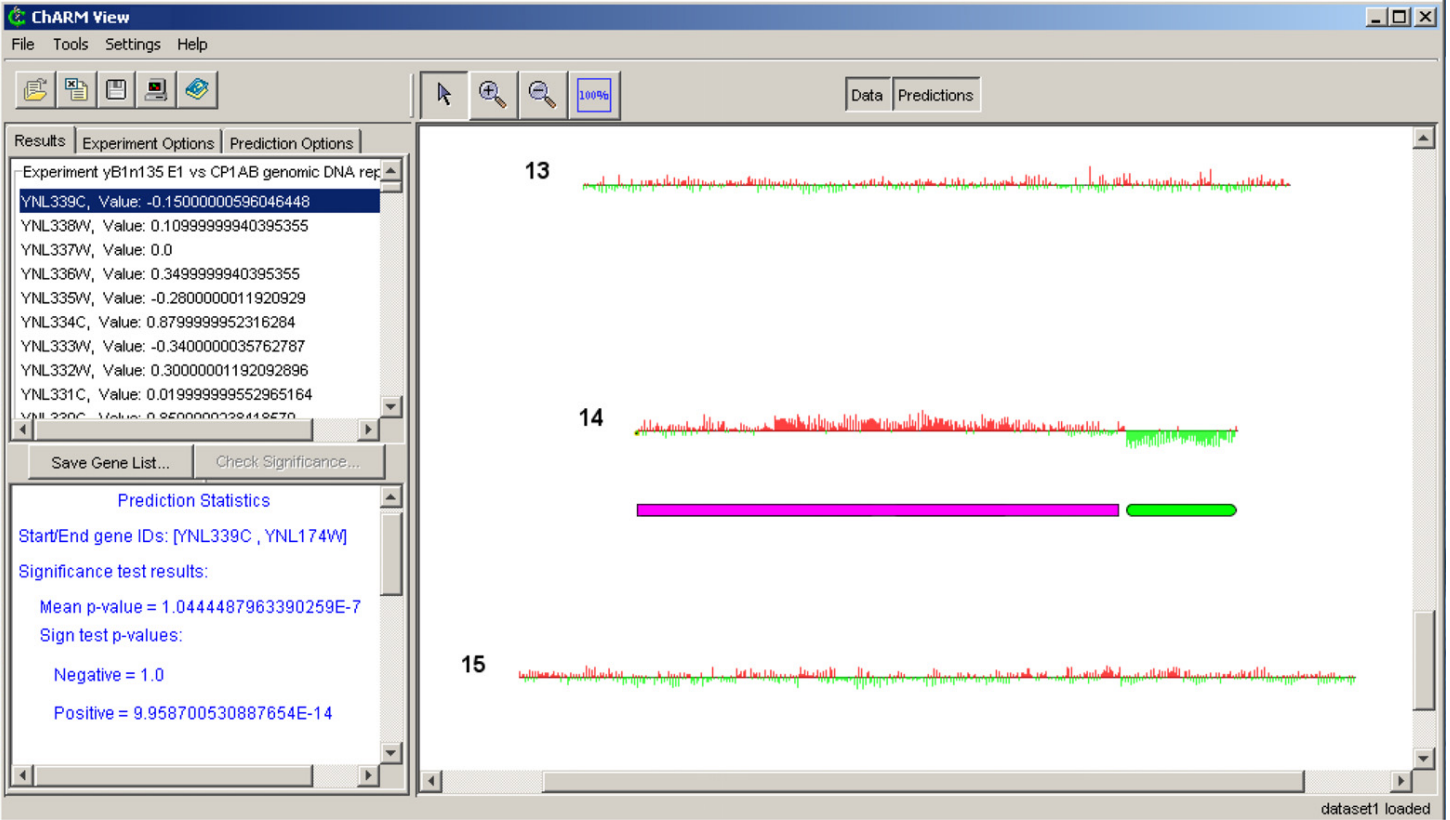
**Screenshot of ChARMView applied to *S. cerevisiae *array CGH data. **Screenshot of ChARMView analysis of *S. cerevisiae *molecular evolution experiments data from Dunham *et al *[5]. The right panel displays array CGH data arranged in the order of chromosomal position and amplification (red) and deletion (green) predictions. The left panel displays information for the selected region, including gene names and values and statistics for the selected amplification prediction.

We have applied ChARMView to a number of array CGH and gene expression datasets, including data derived from both *Saccharomyces cerevisiae *and human experiments. Here we present an example application of our software to array CGH data from experimental evolution experiments in which eight strains of budding yeast were analyzed for chromosomal copy number changes after 100–500 generations of growth in glucose-limited chemostats [5]. Dunham *et al*. confirmed aneuploidy regions identified by array CGH through pulsed-field gel electrophoresis, thus creating a standard for assessing our results. Our method identified all 12 of the confirmed aneuploidies and two additional regions of bias. The novel regions identified by our method correspond to biases smaller than the ones identified by Dunham *et al*. [5] and may reflect aneuploidy present in a subset of cells in the population or may be due to a hybridization artifact. Further laboratory experiments are required to further evaluate these predictions. Figure 5 shows a screenshot of our application upon finishing automated statistical analysis of one of these experiments.

**Figure 6:**
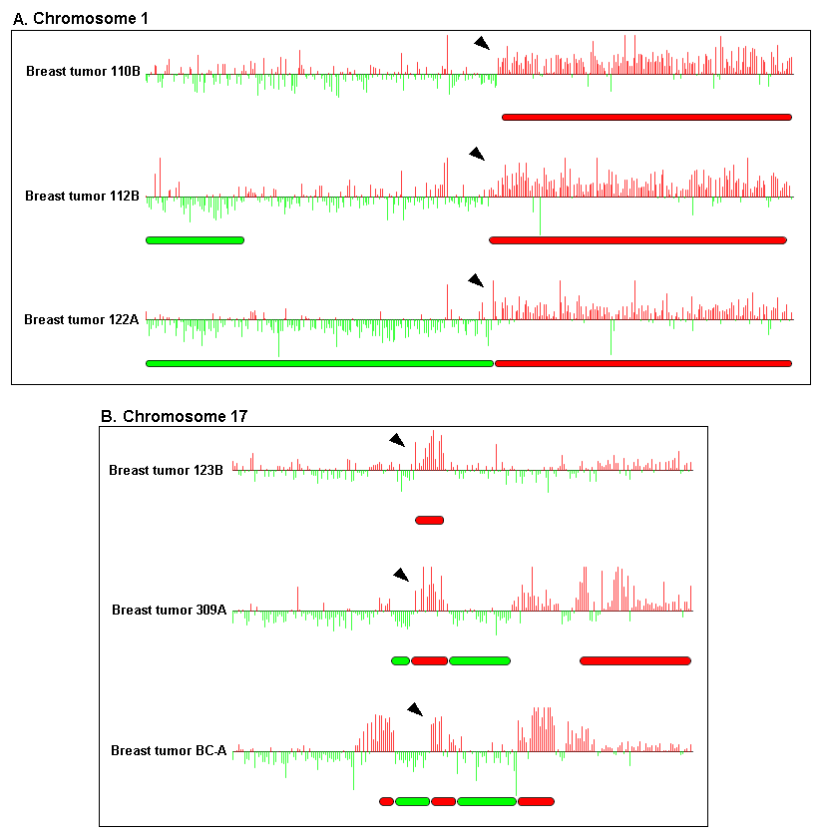
**Predicted amplifications and deletions on breast cancer array CGH data. **ChARMView automated predictions on three breast tumor array CGH profiles (110B, 112B, 122A) from chromosome 1 and three profiles (123B, 309A, and BC-A) from chromosome 17 of [18]. The predicted chromosome 1 breakpoints (identified by arrows in Figure 6A) are 3, 1, and 0 probes from the centromere. The predicted chromosome 17 amplification common to all three profiles (identified by arrows in Figure 6B) includes 7 genes known to be typically amplified with the *ERBB2 *locus. All visible predictions have Bonferroni-corrected p-values less than .05 for both mean and sign significance tests. See Table 2 for a complete list of breakpoint predictions for each of the results pictured.

We also present two specific instances from an array CGH breast cancer study where ChARMView can be used to visualize and accurately predict breakpoints of known amplifications. Figure 6A illustrates the results of ChARMView's automated statistical analysis on chromosome 1 array CGH profiles of three different breast tumor samples (110B, 112B, 122A) from [18]. The entire q arm of chromosome 1 is known to frequently amplified in breast cancer (typically observed in approximately 50–60% of tumors [20,21]). Thus, we expect the amplications here to begin at or near the centromeric end of the q arm. ChARMView predicts breakpoints 3, 1, and 0 probes from the centromeric end of the q arm for samples 110B, 112B, and 122A respectively.

ChARMView can also be used to accurately find much smaller regions of amplification or deletion and the associated breakpoints. Figure 6B illustrates this capability on chromosome 17 array CGH profiles of three breast tumor samples (123B, 309A, and BC-A) from [18]. An amplicon frequently associated with breast tumors includes the *ERBB2 *oncogene at 17q12. While breakpoints identified in individual tumors vary, recent studies have identified a group of 7 genes surrounding the *ERBB2 *locus that are commonly amplified, including *NEUROD2*, *MLN64, PNMT, ERBB2*, *GRB7*, *ZNFN1A3*, and EST 48582 [22,23]. ChARMView's amplification predictions for the three tumor profiles shown include 15, 18, and 13 probes respectively, all of which span the 7-gene region previously identified. All predictions shown in Figure 6 have Bonferroni-corrected p-values less than .05 for both mean and sign significance tests. Complete lists of predicted breakpoints for both chromosome 1 and chromosome 17 amplicons are included in Table 2.

**Table 2:** Examples of predicted breakpoints in breast tumor aCGH case study. Listing of Unigene IDs corresponding to predicted breakpoints for ChARMView results pictured in Figures 6A and 6B. The Unigene ID and gene name are the first and last markers included in the predicted amplification. All results listed have Bonferroni-corrected less than p-values of .05 for both mean and sign significance tests.

**Tumor sample**	**Chrom.**	**Predicted start breakpoint**	**Adjacent gene (in amplicon)**	**Predicted end breakpoint**	**Adjacent gene (in amplicon)**
110B	1	Hs.15871	ACP6	Hs.7395 (last marker)	TFB2M
112B	1	Hs.59889	HMGCS2	Hs.7395 (last marker)	TFB2M
122A	1	Hs.381235	SEC22L1	Hs.7395 (last marker)	TFB2M
123B	17	Hs.97477	LYZL6	Hs.276916	NR1D1
309A	17	Hs.73817	CCL18	Hs.267871	PTRF1
BC-A	17	Hs.635	CACNB1	Hs.2340	HAP1

### Usage

ChARMView can be downloaded at  and run on virtually any platform if the J2SE Java Runtime Environment version 1.4.2 or greater is present. A brief overview of the primary features of the software follows.

#### Loading data

ChARMView accepts all types of data from any organism provided that the features can be ordered on a set of linear chromosomes. Input files must be tab-delimited, specifically in the commonly-used .pcl format. Chromosome labels and position must be included in the input file unless the organism type is *Saccharomyces cerevisiae *or human with ORF or Unigene identifiers, which ChARMView is able to order without coordinates.

#### Viewing data

Figure 5 shows a typical ChARMView screenshot upon loading data and statistical analysis. The data display is zoomable and selectable with mouse-overs for identification of experiments and individual genes. Zoom features include standard single-click magnification, zoom to rectangle, and zoom reset (fit to screen) capabilities. When one or more gene or probe data points are selected, identifiers and associated annotation are displayed in the "Results" tab, which appears adjacent to the display panel. This allows users to select regions of interest on the display panel and retrieve lists of genes or probes within these regions. Additionally, any number of experiments may be viewed simultaneously by toggling the corresponding checkboxes in the "Experiment Options" tab, also adjacent to the display panel.

#### Analyzing data

ChARMView supports two different modes of analysis. The first employs the automated edge-finding algorithm discussed in Myers *et al*. [16] followed by statistical analysis. The second mode is for testing user-selected regions of data and only evaluates the statistical significance of the chosen region. Both methods of analysis rely on two tests of statistical significance: a mean-based permutation test, and a one-sample sign test. Details of both tests are discussed above and in Myers *et al*. [16]. P-values for these tests are reported for all regions found by the automated approach or selected by the user. Figure 5 displays a typical view of statistical results for a single experiment. Note that the red and green rectangles below the data correspond to regions of predicted aberration. The p-value cutoff at which results of the statistical analysis appear in the display panel can be adjusted by applying p-value filters provided in the "Prediction Options" tab adjacent to the display panel.

A p-value filter consists of a logical combination of the mean permutation test and/or the one-sample sign test and real-valued cutoffs for each test. These combinations specify how the selected p-value cutoffs will be used to deem statistical significance. For instance, one possible p-value filter is "Sign AND Mean Tests" with Sign p-value cutoff of 0.001 and Mean p-value cutoff of 0.01, which will result in only predictions with both Bonferroni corrected sign p-values of less than .001 and mean p-values of .01 being displayed. The Bonferroni corrected p-value is obtained by multiplying the raw p-value from both significance tests by the number of regions tested for that chromosome. Another possibility is to apply "Sign OR Mean Tests", which results in a prediction being displayed if at least one of these criteria is met at the specified significance level. While we recommend the "Sign AND Mean Test" option for general use, other combinations may be useful under certain circumstances. Users can select any displayed prediction, which results in the genes or probes and associated annotation in that particular region to be displayed in the "Results" tab adjacent to the display panel (Figure 5).

#### Exporting results

Publication quality images can be exported in multiple formats at any stage of the visualization. This includes images of exclusively raw data, results of statistical analysis, or combinations of these. In addition, predictions resulting from automated or manual statistical analysis can be exported in tab-delimited text form with the associated gene or probe identifiers and corresponding p-values. A p-value filter similar to that described in "Analyzing data" can be applied to all exported results to allow full user control over which predictions are included. Finally, lists of genes or probes for any object selected on the display panel can also be exported to text files to facilitate immediate analysis of regions identified by manual inspection.

#### Command-line usage

ChARMView can also be used in command-line mode to make automated predictions of amplification or deletions. This command-line feature can be used by invoking ChARMView as follows:

java -Xmx300m -jar ChARM.jar

-inputFile <input-file>

-outputFile <output-file>

-organismType <organism-type>

-meanPvalCutoff <mean-pvalue-cutoff>

-signPvalCutoff <sign-pvalue-cutoff>

-sigTestType <significance-test-type>

The possible organism types, which determine reference chromosomal coordinates, are: 1, *Saccharomyces cerevisiae*; 2, human; 3, other (user-provided coordinates). Possible significance test options include: 1, mean AND sign tests; 2-mean OR sign tests; 3, mean test only; 4, sign test only. When run in command-line mode, ChARMView outputs all predicted regions of amplification and deletion meeting the specified significance level.

## Conclusion

We have developed ChARMView, a statistical visualization system for analysis and discovery of genomic aberrations. Our system can analyze various types of genomic data, including gene expression and array CGH microarray data, for a variety of organisms, and has been developed to facilitate both manual discovery through powerful visualization as well as automated prediction through robust statistical analysis. ChARMView can identify and visualize even small copy number changes, and is sensitive enough to detect aneuploidies in mixed populations of cells. This combination makes ChARMView uniquely effective for identifying subtle trends, recurring aberrations in sets of experiments, and pinpointing functionally relevant copy number changes. Thus, this system is effective for identification of aneuploidies in cancer studies and molecular evolution experiments, as well as for routine analysis of microarray data for special biases.

## Availability and requirements

Project name: ChARMView

Project homepage: 

Operating system(s): Platform independent

Programming language: Java

Other requirements: J2SE Java Runtime Environment 1.4.2 or higher

License: GNU GPL

Any restrictions to use by non-academics: None

## Authors' contributions

CLM developed the methodology, the software components, and performed case studies on example datasets. XC, together with CLM, developed an early version of the software. CLM and OGT drafted the manuscript. OGT conceived of the idea for ChARM View and directed the development. All authors read and approved the final version of the manuscript.
